# Long-lasting benefit on multimodal treatment combining osimertinib and stereotaxic radiotherapy for metastatic non-small cell lung cancer with the EGFR exon 20 insertion 773-774 HVdelinsLM: a case report

**DOI:** 10.3389/fonc.2023.1143775

**Published:** 2023-07-03

**Authors:** Aurélie Louvet, Natasha Honoré, Anne-France Dekairelle, Cédric Van Marcke, Jean-Charles Goeminne

**Affiliations:** ^1^ Department of Medical Oncology, Centre Hospitalier Universitaire de l’ Université Catholique de Louvain (CHU UCL) Namur site Sainte-Elisabeth, Namur, Belgium; ^2^ Department of Medical Oncology, King Albert II Cancer Institute, Cliniques Universitaires Saint-Luc, Brussels, Belgium; ^3^ Institut de Recherche Expérimentale et Clinique (IREC), Pole of Medical Imaging, Radiotherapy and Oncology (MIRO), Université Catholique de Louvain (UCLouvain), Brussels, Belgium; ^4^ Department of Genetics, Cliniques Universitaires Saint-Luc, Brussels, Belgium

**Keywords:** non-small cell lung cancer, EGFR, exon 20, tyrosine kinase inhibitor, osimertinib, stereotaxic radiotherapy, multimodal

## Abstract

A non-small-cell-lung-cancer patient with cerebral metastasis presenting an atypical exon 20 mutation in the EGFR gene had a long-lasting tumor cotrol on mulimodal treatment with osimertinib and stereotaxic radiotherapy on oligoprogressing lesions. Most exon-20 mutations are resistant to first, second and third generation EGFR-directed TKI. This case was discussed on our molecular tumour board. As the more specific exon-20 targeted therapies were not yet available and as sporadic short responses on the third generation EGFR-directed TKI, osimertinib had been described, the patient started osimertinib. She had a prolonged tumoral response on Osimertinib. The patient is still asymptomatic up to 32 months after initiating the medication. This case confirms that not all exon20 EGFR mutations are equal to osimertinib and that the localization of the exon 20 insertion mutation is probably important to consider when treating EGFR mutated NSCLC. The long-term clinical benefit can be maintained through stereotactic radiotherapy on focal progressive lesions.

## Introduction

Non-small cell lung cancer (NSCLC) accounts for 85% of lung cancer cases (1) and is frequently characterized by the presence of an oncogenic event that drives malignancy. In the last decade, successful targeted treatments have been developed for specific pathogenic alterations in several genes such as *EGFR*, *ALK*, *ROS*, *BRAF*, *MET*, *RET*, *ERBB2, NTRK* and *KRAS*. These targeted treatments against oncogenic mutations or genomic rearrangements offer patients more effective treatments with fewer side effects and longer-lasting responses than with conventional chemotherapies. Furthermore, NSCLC driven by activating mutations are often less sensitive to immunotherapy (2). Most of these mutations are found in the non-squamous NSCLC, especially in never-smokers or light-smokers.

NSCLC cases harbouring *EGFR* driver mutations represent 10 - 20% of all lung cancer cases in Europe (3). Approximately 90% of *EGFR* gene mutations are insertions or deletions in exon 19 or point mutations (mainly L858R) in exon 21. These mutations confer high sensitivity to the first- and second-generation EGFR tyrosine kinase inhibitors (TKI) erlotinib, gefitinib and afatinib. Meanwhile, the third-generation EGFR TKI osimertinib has shown superior efficacy in the first line as compared to the first-generation molecules (4) and is able to overcome resistance to these molecules induced by the *EGFR* T790M mutation in exon 20 (5).

Approximately 10 - 15% of EGFR-driven NSCLC have uncommon *EGFR* mutations (6), among which exon 20 in-frame base pair insertions represent the largest subgroup (7). Until recently, clinical data regarding treatment outcomes with EGFR TKI were scarce, as uncommon *EGFR* mutations were excluded from studies testing the efficacy of anti-EGFR treatments. Nevertheless, most *EGFR* exon 20 insertions are considered resistant to first- and second-generation anti-EGFR TKIs (8). Small studies and case reports suggest limited activity of the third-generation anti-EGFR TKI, osimertinib on NSCLC with *EGFR* exon 20 insertions (9, 10), with lower response rates and shorter progression-free survivals compared to NSCLC with EGFR exon 19 or exon 21 mutations.

We present a case of a metastatic NSCLC driven by an *EGFR* exon 20 in-frame insertion with a long-term response to osimertinib.

## Case report

In February 2020, a 68 years-old woman with an ECOG performance status of zero presented with dry cough and hemoptysis. She never smoked and had a medical history of arterial hypertension, an adrenal non-progressing hyperplasia, a gastric bypass and a cholecystectomy. In the familial oncological history, we note an adrenal carcinoma in two brothers and a metastatic neoplasia of unknown origin in her father. A genetic testing for germinal mutations was not performed.

Chest computed tomography (CT) revealed pulmonary infiltration of the left lower lobe, with small mediastinal lymph nodes.

Bronchial left lower lobe biopsy revealed a lung adenocarcinoma, TTF1 (+), P40(-). The NGS (QiaSeq targeted DNA Panel CDHS-15662Z-617) testing for oncogenic driver mutations showed a p.773-774HVdelinsLM variant located in exon 20 of the *EGFR* gene and a TP53 co-mutation (**Table 1**). ALK and ROS immunochemistry staining were negative, whereas PD-L1 expression was estimated to be 90% with the 22C3 antibody (Dako PharmDx assay).

**Table 1:** identified mutations (nomenclature HGVS - reference hg19).

Gene	p./c.	Exon	Allelic frequency of the variant (VAF)	Biological classification
***EGFR: NM_005228***	c.2318_2320delinsTCA p.(His773_Val774delinsLeuMet) p.(H773_V774delinsLM)	20	52,4%	Probably pathogenic in NSCLC
***TP53: NM_000546***	p.(?) p.(?) c.376-2A>C	5	26.7%	Probably pathogenic

The work-up was completed by a PET-CT and a brain MRI. Three asymptomatic brain metastases were observed on MRI, leading to the diagnosis of a cT3N2M1 lung adenocarcinoma. Afterwards, the disease was evaluated every three months by chest and abdominal CT, brain MRI and PET-CT on progression of single lesions.

In the multidisciplinary tumour board first-line immuno-chemotherapy was initially considered as an appropriate treatment option, because specific exon-20 targeted therapies were not yet available at the time of diagnosis and single agent immunotherapy is considered as less effective in EGFR-mutated lung carcinomas even with high PDL1 expression.

The treatment options were discussed at our multidisciplinary molecular tumor board (MTB). As a case report reported a favorable response in a patient presenting this specific mutation (11), our patient was put on osimertinib treatment at a daily dose of 80mg.

She noted significant regression of coughing after one month of treatment.

At the first tumor assessment after 3 months of treatment, there was a partial response according to the RECIST 1.1 criteria with a moderate decrease in the pulmonary tumor infiltration, a significant regression of the brain metastases and disappearance of the cerebral perilesional oedema, the main lesion decreasing from 12,5 x 13 mm to 7.5 x 8 mm.

Stereotaxic radiotherapy of an occipital 8 mm brain lesion was performed after 8 months, as some asymptomatic perilesional edema had reappeared without change in size of the lesion.

**Figure 1:**
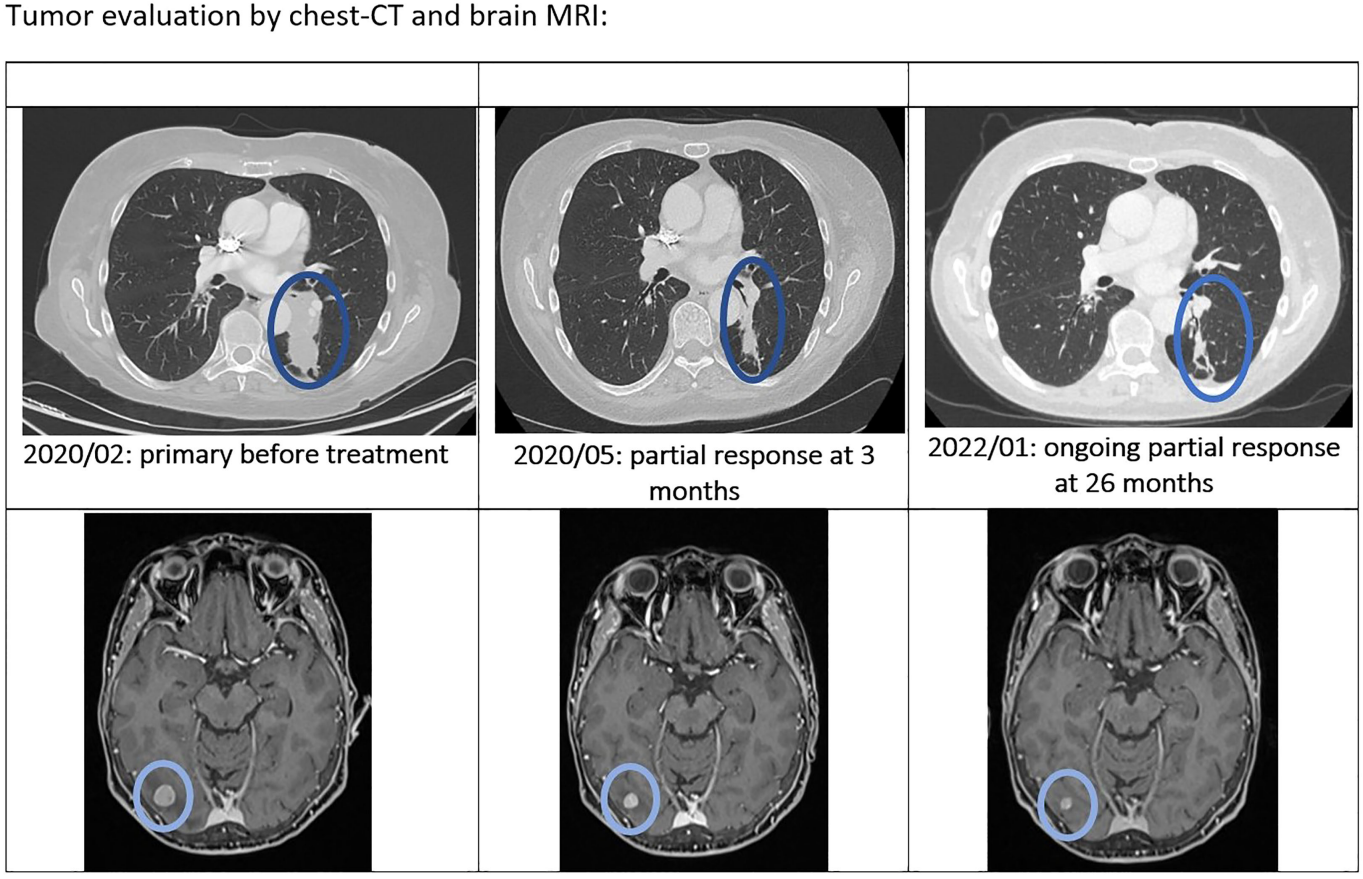
Tumor evaluation by chest-CT and brain MRI.

After 20 months of treatment, stereotaxic radiotherapy was performed on a second frontal brain lesion that increased discretely from 3 to 5 mm. Osimertinib treatment was continued as there was no progression of the other tumor locations (**Figure 1** - April 2022).

**Figure 2:**
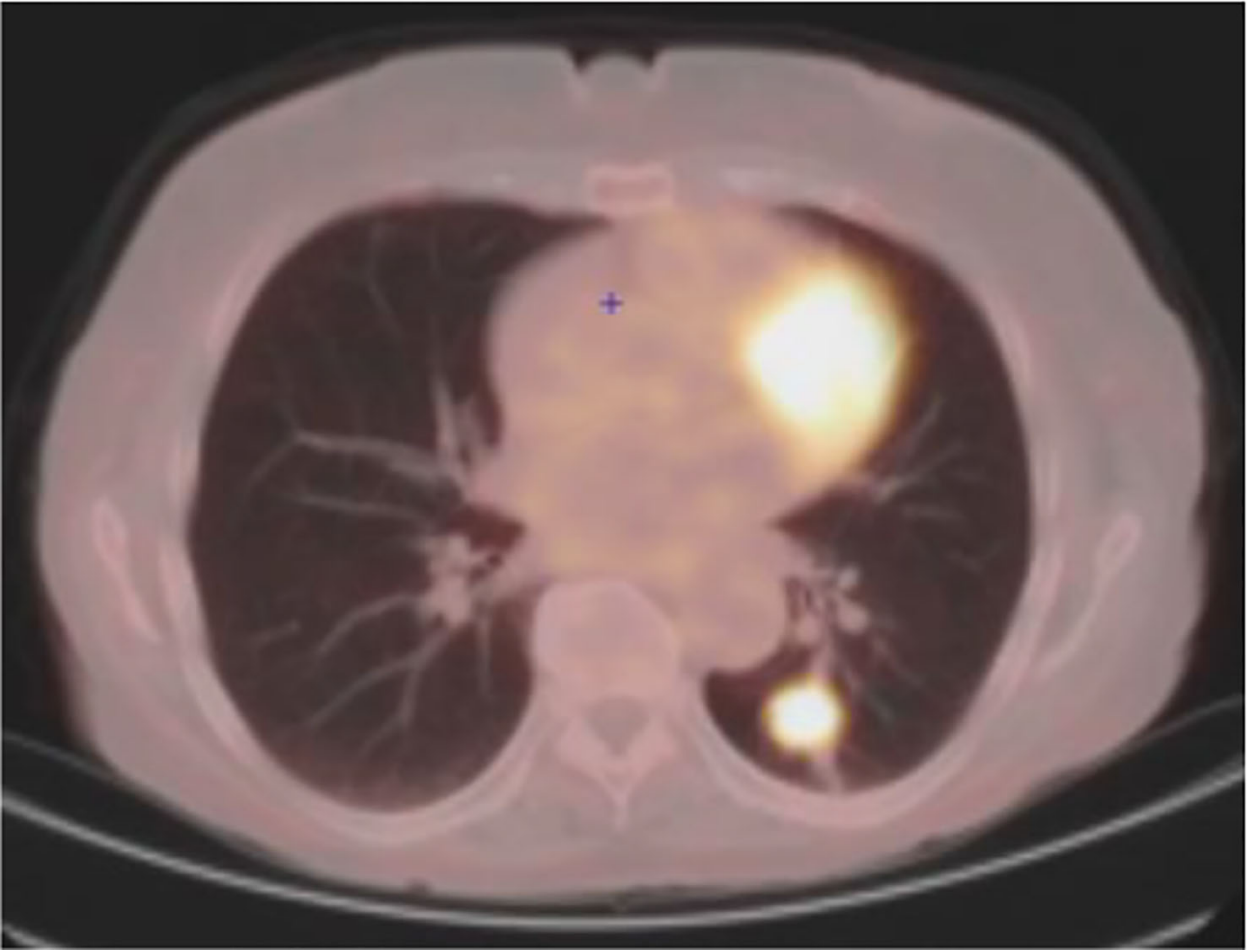
August 2022: oligometastatic lung progression.

In August 2022, 30 months after the initiation of osimertinib, the patient presented a focal asymptomatic progression in the main left thoracic lesion, without signs of other extracerebral or cerebral progression, as confirmed by PET-scan (**Figure 2**) and brain MRI. The thoracic progression was treated by stereotaxic radiotherapy, and osimertinib was continued.

At the last tumor evaluation on April the 26th 2023, 38 months after diagnosis, the patient is still on Osimertinib and free of symptoms, without signs of progression on brain MRI and thoraco-abdominal CT.

Timeline of the combined targeted treatment and stereotaxic radiotherapy on focal progressing lesions

**Table:** 

February 2020, diagnosis of a metastatic lung adenocarcinoma cT3N2M1, EGFR exon 20 mutated - Initiation of Osimertinib treatment 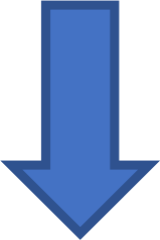
May 2020, partial response of brain and lung lesions 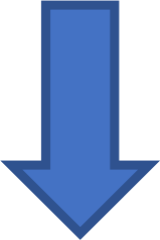
October 2020, stereotaxic radiotherapy on a cerebellar lesion with reappearance of mild edema 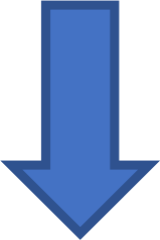
October 2021, stereotaxic radiotherapy on a small progressing cerebral frontal lesion 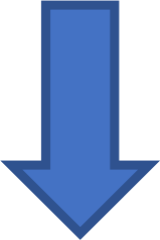
August 2022, stereotaxic radiotherapy on an oligo-progressive thoracic lesion 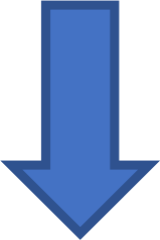
April 2023, no signs of cerebral or extra-cerebral progression, the patient is still asymptomatic

## Discussion

This case report describes the long-term efficacy of the third generation anti-EGFR TKI osimertinib through a multimodality treatment in a patient with metastatic lung adenocarcinoma with the p.773-774HVdelinsLM in-frame insertion located in exon 20 of the *EGFR* gene. The choice to prescribe Osimertinib rather than chemotherapy follows multiple discussion with our oncogeneticists within their mutational tumor board. The patient’s particular mutation was considered oncogenic and potentially targetable after review of the literature, where a case report reported a 12-months lasting response to osimertinib in a patient with NSCLC with the same 773-774HVdelinsLM *EGFR* alteration after failure of gefitinib. Noteworthy bevacizumab was combined with Osimertinib in this patient (11).

**Figure 3:**
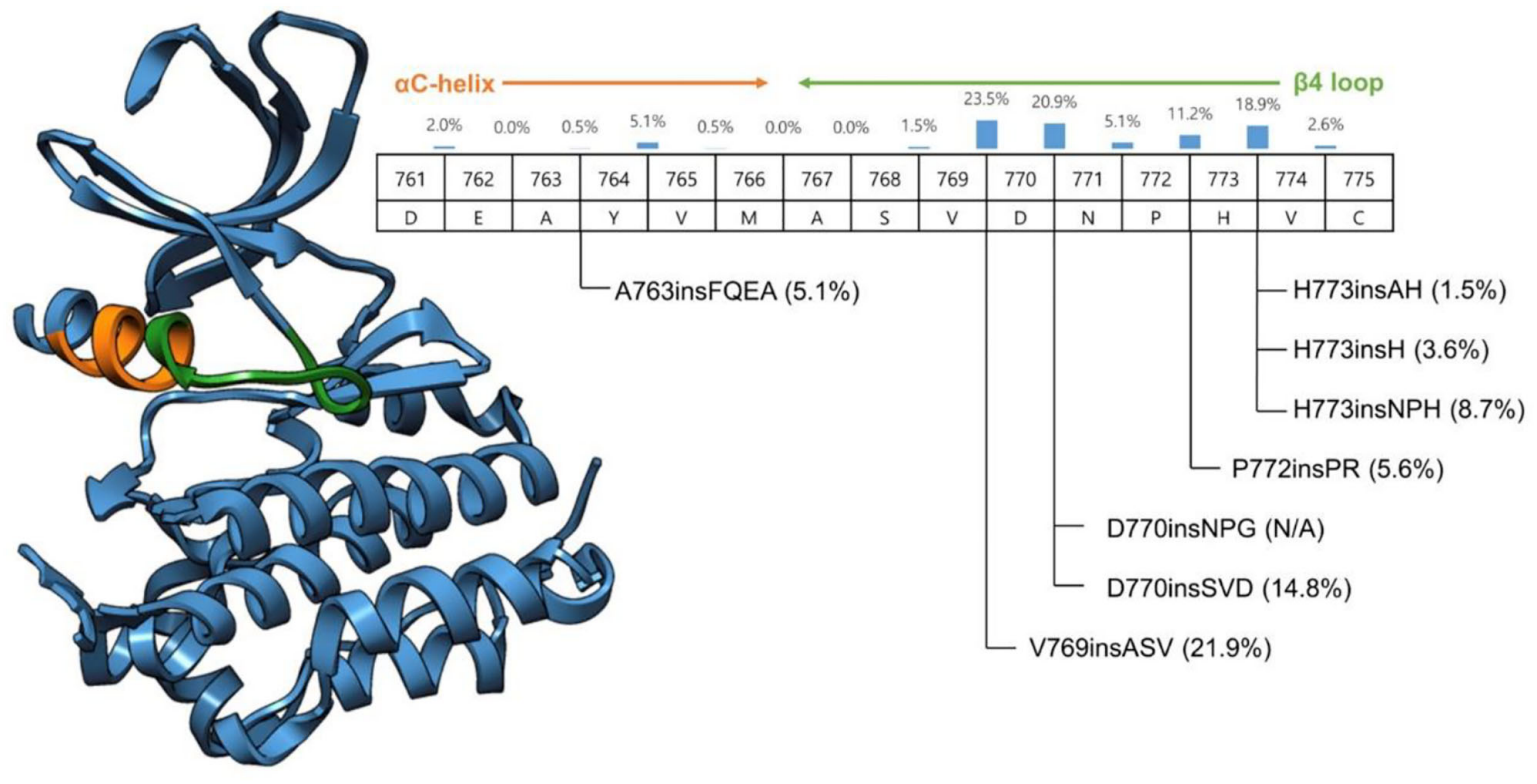
Crystallographic structure of the EGFR protein.

*Exon* 20 in-frame base-pair insertions affect the region coding for the immediate environment of the C-helix domain of the protein, thereby permanently stabilizing the active state of EGFR. Nevertheless, genomic and crystallographic studies have revealed that they belong to a heterogeneous group. Insertions within the C-helix domain (amino acids 761-766) appear to remain sensitive to first generation EGFR TKIs *in vitro* and *in vivo*, whereas insertions located in the region coding for the loop immediately following the C-helix (amino acids 767-775) also modify the drug-binding pocket of the protein, thereby drastically reducing its affinity for EGFR inhibitors (12, 13). The insertion described in our patient modifies the loop following the C-helix domain (**Figure 3**). The prolonged and ongoing activity of osimertinib in this patient was unexpected.

Although osimertinib has an inequivalently higher affinity and irreversible binding capacity to EGFR compared to first- and second-generation EGFR TKIs, their mechanisms of action are by nature similar (14). Therefore, exon 20 base insertions are generally considered as intrinsically resistant to osimertinib, and in clinical practice, until recently, chemotherapy was the preferred treatment choice in this setting (15). Deeper pre-clinical studies have shown a heterogeneous response to osimertinib depending on whether the insertion is within or after the C-helix domain (16). In phase I/II studies including patients with *EGFR* exon 20 uncommon insertion-mutation post C-helix domain, osimertinib failed to show durable clinical responses. A large cohort study showed heterogeneous responses to anti-EGFR TKIs, depending on the localization of the alteration in exon 20. However, the insertions differed from our clinical case (17). In addition to the localization of the alteration with regard to the C-helix domain, other factors might also explain the heterogeneity in the response to anti EGFR TKIs: the addition of different amino-acids may change the insertion pocket of osimertinib in different ways. Some mutations can co-occur with other anomalies in other genes, which could change the response to therapy and confer primary resistance. When considering the specific alteration of our patient, one cohort study, reporting limited responses to osimertinib in *EGFR* exon 20 insertion-mutation NSCLC, included two cases with the 773-774HVdelinsLM alteration: one had a short stable disease (progression-free survival (PFS) of 3.2months), and one had a partial response with a PFS of 8.3 months (18).

In this patient a TP53 co-mutation was detected. This co-mutation could affect primary sensitivity as well as acquired resistance to EGFR-TKIs as suggested by an observational longitudinal Spanish cohort study (19) and by *in vitro* on EGFR-mutated cell-cultures (20).

Recently two new TKIs specifically targeting *EGFR* exon 20 in-frame insertions, mobocertinib (21) and poziotinib (22) and a bi-specific monoclonal antibody targeting MET-EGFR, amivantamab (23), showed very encouraging clinical activity in *EGFR* exon-20 insertion-mutation NSCLC, although they are probably less well tolerated compared to Osimertinib. Divergent responses on amivantamab were observed, depending on the location of the alteration: helical region (overall response rate (ORR) of 100%), near loop (ORR of 41%), far loop (ORR of 25%). Similar findings were observed with the efficacy of anti-angiogenic therapy dependent on the EGFR ex20ins loop location (24).

The EGFR structure has been studied extensively. Based on retrospective patient reports, *EGFR* mutations were separated into four subgroups. The subgroup characterized by structural changes in the EGFR protein could better predict sensitivity to EGFR inhibitors than traditional exon-based groups. In the future treatment could be guided by a more protein structure-based approach (25).

These results should still be considered hypothesis generating. Further exploration in ongoing studies will demonstrate whether the localization of the exon 20 insertion mutation is a key element to consider when treating *EGFR* mutated NSCLC.

After the patient initiated therapy, several reports confirmed the potential benefit of Osimertinib on other unusual EGFR (exon 20 insertions excluded), mainly G719X (30%), L861Q (26).

In our patient long-term clinical benefit was maintained through stereotaxic radiotherapy on focal progressive lesions, while keeping the patient on osimertinib. The global response to Osimertinib could be shorter than that observed with common exon 19 mutations, although the first PFS is difficult to determine in this patient because the two cerebral stereotaxic radiotherapy treatments were performed before the patient could be considered as progressive according to RECIST criteria. Local therapy for oligoprogressive disease on EGFR directed therapy can often offer a prolonged tumoral control (27).

## Conclusion

Our case report describes a long-lasting major clinical benefit on third generation anti-EGFR antibody Osimertinib, with local consolidative therapy on oligoprogressive lesions.

It confirms that not all exon20 EGFR mutations are equal to osimertinib and that the localization of the exon 20 insertion mutation is probably important to consider when treating EGFR mutated NSCLC.

The analysis of recently published early phase clinical studies and case series suggests that it is important to consider not only the mutated gene and exon, but also the specific localization of the alteration, it can affect significantly the response to a targeted drug. Hopefully, ongoing studies will confirm whether exon 20 *EGFR* mutations should be clustered by their localization in the C-helix domain to better predict the treatment effects. This also highlights the importance of pre-clinical work and specific protein models of specific genomic abnormalities.

## Data availability statement

The datasets presented in this article are not readily available because of ethical/privacy restrictions. Requests to access the datasets should be directed to the corresponding author.

## Ethics statement

Written informed consent was obtained from the individual(s) for the publication of any potentially identifiable images or data included in this article.

## Author contributions

All participated equally to the report. All authors contributed to the article and approved the submitted version.
